# Early Lipid Raft-Related Changes: Interplay between Unilateral Denervation and Hindlimb Suspension

**DOI:** 10.3390/ijms22052239

**Published:** 2021-02-24

**Authors:** Irina G. Bryndina, Maria N. Shalagina, Vladimir A. Protopopov, Alexey V. Sekunov, Andrey L. Zefirov, Guzalia F. Zakirjanova, Alexey M. Petrov

**Affiliations:** 1 Department of Pathophysiology and Immunology, Izhevsk State Medical Academy, Kommunarov St. 281, Izhevsk 426034, Russia; i_bryndina@mail.ru (I.G.B.); uvula@mail.ru (M.N.S.); vladimirvst@yandex.ru (V.A.P.); d1key@inbox.ru (A.V.S.); 2 Institute of Neuroscience, Kazan State Medical University, Butlerova St. 49, Kazan 420012, Russia; zefiroval@rambler.ru (A.L.Z.); gffysiology@gmail.com (G.F.Z.); 3 Laboratory of Biophysics of Synaptic Processes, Kazan Institute of Biochemistry and Biophysics, Federal Research Center “Kazan Scientific Center of RAS”, P. O. Box 30, Lobachevsky St. 2/31, Kazan 420111, Russia

**Keywords:** skeletal muscle, disuse, denervation, deafferentation, capsaicin, lipid rafts, neuromuscular junction, acid sphingomyelinase, ceramide, apoptotic proteins

## Abstract

Muscle disuse and denervation leads to muscle atrophy, but underlying mechanisms can be different. Previously, we have found ceramide (Cer) accumulation and lipid raft disruption after acute hindlimb suspension (HS), a model of muscle disuse. Herein, using biochemical and fluorescent approaches the influence of unilateral denervation itself and in combination with short-term HS on membrane-related parameters of rat soleus muscle was studied. Denervation increased immunoexpression of sphingomyelinase and Cer in plasmalemmal regions, but decreased Cer content in the raft fraction and enhanced lipid raft integrity. Preliminary denervation suppressed (1) HS-induced Cer accumulation in plasmalemmal regions, shown for both nonraft and raft-fractions; (2) HS-mediated decrease in lipid raft integrity. Similar to denervation, inhibition of the sciatic nerve afferents with capsaicin itself increased Cer plasmalemmal immunoexpression, but attenuated the membrane-related effects of HS. Finally, both denervation and capsaicin treatment increased immunoexpression of proapoptotic protein Bax and inhibited HS-driven increase in antiapoptotic protein Bcl-2. Thus, denervation can increase lipid raft formation and attenuate HS-induced alterations probably due to decrease of Cer levels in the raft fraction. The effects of denervation could be at least partially caused by the loss of afferentation. The study points to the importance of motor and afferent inputs in control of Cer distribution and thereby stability of lipid rafts in the junctional and extrajunctional membranes of the muscle.

## 1. Introduction

Skeletal muscle atrophy is the loss of muscle mass caused by conditions such as disuse, neuromuscular disorders, catabolic conditions and aging. Muscle disuse is one of the most common cause of muscle wasting. Several methods, including hindlimb suspension (HS) and denervation, are widely used to simulate muscle disuse in experiments on rodents [1,2]. Although both HS and denervation lead to the development of muscle atrophy, there is evidence that, in some aspects, they induce different underlying pathophysiological mechanisms [3]. Accordingly, early events in response to HS and denervation can be crucial for triggering different chains of pathological cascade. Revealing these initial events may provide information indispensable for developing specific therapeutic approaches.

Short-term HS leads to marked changes in the plasma membrane properties, particularly disruption of lipid rafts in the skeletal muscle [4,5]. Lipid rafts represent dynamic membrane microdomains enriched in cholesterol (Chol) and sphingolipids. Due to their unique biochemical properties, lipid rafts participate in compartmentalization of signaling proteins and their complexes as well as in membrane dynamics including processes of exo- and endocytosis. In skeletal muscle, lipid rafts are highly abundant at the pre- and postsynaptic regions and many essential for neuromuscular transmission proteins reside in these microdomains [4,6,7,8]. Additionally, a crucial for lateral force transmission molecular link between the intracellular cytoskeleton and the extracellular matrix is dependent on lipid raft integrity in skeletal muscle [9]. Defects in a muscle lipid raft stabilizing protein caveolin 3 causes limb-girdle muscular dystrophy, rippling muscle disease and distal myopathy [10]. Accordingly, lipid rafts instability could be an atrophy-promoting early event in disused skeletal muscle. 

The mechanism of HS-induced lipid raft disruption is associated with increased cleavage of a key raft component sphingomyelin by acid sphingomyelinase (aSMase) [5]. Additionally, there is evidence that ceramide (Cer), a backbone molecule of sphingolipids, accumulates in unloaded soleus muscle [11,12,13], particularly in the plasma membrane raft and nonraft regions including neuromuscular junctions [5,8,14]. A functional inhibitor of aSMase clomipramine attenuates some effects of muscle disuse: lipid raft disassembly, loss of membrane asymmetry, decrease in sphingomyelin content, Cer accumulation in lipid rafts and formation of Cer-enriched membrane domains [5,14,15]. Given that Cer is an important messenger of inflammation and excess Cer has proapoptotic functions and may deteriorate insulin sensitivity in skeletal muscles [16], Cer accumulation at early stage of muscle disuse could contribute to triggering of atrophy. Furthermore, Cer can displace Chol from raft membrane, leading to decrease in the lipid raft integrity and dissociation of caveolin from the raft [17,18]. Despite discovering the early lipid-related effects of HS, changes in lipid raft integrity and Cer levels in the raft fraction in response to denervation have not been studied yet. However, upregulation of Cer and its derivative glucosylCer has been demonstrated in homogenates from denervated soleus muscles [19,20,21].

Another poorly studied aspect, important for understanding mechanisms of disuse muscle atrophy, is interaction between the effects of HS and denervation when they coexist. Tischler et al. [22] found that glucose uptake disturbance in denervated-unweighted muscles did not differ from those in denervated-weight-bearing muscles; therefore, they concluded that the effects of denervation must be independent of leg posture. Kuiawa et al. [23] have found that a tendency of decline in muscle satellite cell activity due to immobilization was similar to those in denervated soleus muscle; moreover, immobilization after denervation aggravated some of the ultrastructural alterations (i.e., decrease in nucleolus volume fraction, in ribosome-like structures number and in number of mitochondria) in the cells studied. In the experiments of Di Maso et al. [24], the enhancement of the fast IIb myosin heavy chain expression in rat soleus muscle due to 2-week HS combined with T_3_ treatment was prevented with denervation. The authors emphasized that normal innervation is essential for inducing the unique expression of the fast IIb myosin heavy chain in a slow muscle in response to HS. Ohira with coauthors [25] studied the effects of 10-day HS with or without denervation on the cross-sectional area and myosin phenotype in rat soleus and plantaris muscles. Additional denervation in suspended rats had minimal effects on fiber size in the soleus, in opposite to plantaris, in which cross-sectional area further decreased, particularly in fibers expressing only fast myosin heavy chains. Denervation had no further effect on the fiber type composition in either unloaded muscles. Thus, interplay between denervation and HS can be complex and is far from clear. 

Undoubtedly, denervation of hindlimb muscles simulated by transection of the sciatic nerve interrupts both efferent (motor, autonomic) and afferent fibers of the nerve. Picquet et al. [26] have found that, in rats subjected to bilateral dorsal rhizotomy, motor and afferent inputs into the innervated muscle differently affect muscle function in hindlimb unloaded rats. The authors concluded that some alterations in the unloaded muscle (including slow to fast transition) have the motor origin, whereas muscle atrophy and decrease in muscle force are partly the result of deafferentation. Nemirovskaya et al. [27] studied the effects of limb deafferentation caused by unilateral dorsal rhizotomy in rats. Unilateral deafferentation did not affect muscle atrophy in both suspended and freely moving animals, but led to increases in the proportion of soleus muscle fibers containing the slow isoforms of myosin heavy chain. Accordingly, deafferentation and interruption of motor input can have a different contribution to denervation-induced muscle atrophy and affect HS-mediated muscle fiber alterations in a specific way. 

While dorsal rhizotomy eliminates total afferentation from the muscles investigated, the other approach is used for deafferentation without disrupting nerve fibers [28]. Capsaicin (8-methyl-N-vanillyl-*trans*-6-nonenamide; Caps) is an alkaloid found in chili peppers and usually used as an agonist of the transient receptor potential cation channel subfamily V member 1 (TRPV1) also known as the vanilloid receptor 1. These channels are residents of lipid rafts in sensory neurons [29], and location of TRPV1 channels in the lipid rafts regulates functioning of these channels [29,30]. Caps administration leads to strong Na^+^ and Ca^2+^ influx through TRPV1, resulting the membrane depolarization, inactivation of voltage-dependent Na^+^ channels and triggering multiple Ca^2+^-dependent events (i.e., exocytosis) [31]. Prolonged exposure to Caps can damage the TRPV1-expressing sensory neurons due to Ca^2+^ overload [31,32]. Since TRPV1 are preferentially localized to the Caps-sensitive primary afferents (CSPA), topical Caps application allows to selectively inhibit the function of CSPA [33]. Specifically, Caps application onto nerve acts primarily on thin myelinated (Aδ or III type) and unmyelinated (C or IV type) fibers [34]. These fibers can be activated in condition of static contraction of gastrocnemius muscle [35]. Functionally, it has been found that chronic administration of Caps into the rat gastrocnemius muscle (which leads to CSPA deafferentation) is accompanied by increase in the amount of slow-twitch muscle fibers [36], as well as modulation of fatigue and monosynaptic spinal reflex [34]. The effects of partial chemical deafferentation with Caps on HS-induced changes in lipid raft integrity and Cer have not been investigated.

It should be noted that exposure of primary neural afferents to Caps or its analogs triggers an early excitation followed by desensitization. Initially, TRPV1 stimulation leads to release of neuropeptides (substance P, calcitonin gene-related peptide (CGRP) and somatostatin) from CSPA [37]. Then this procedure exhausts the pools of neuropeptides in the terminals of CSPA, thereby preventing further release of those neuropeptides to the tissue in response to a number of stressful stimuli. Release of neuropeptides by CSPA is known as “efferent function of afferent neurons” [28,33]. Note these peptides are highly potent vasodilators and can regulate inflammatory response [38,39]. In addition, CGRP can provoke insulin resistance [38] and affect synthesis of acetylcholine receptor in skeletal muscles [40]. Hence, efferent function of CSPA could be a player in pathogenesis of muscle atrophy. 

The main purpose of the present study is to reveal effects of denervation on Cer levels and lipid raft-related properties in skeletal muscle. Additionally, the interactions between denervation and HS as well as contribution of afferent input in these interactions are of interest. We hypothesized that surgical denervation and deafferentation of sciatic nerve exerts the similar action on Cer accumulation and distribution as well as on membrane properties and proapoptotic activity related with Cer levels. We also believe that denervation and partial chemical deafferentation of the soleus muscle can render the essential effects of short-term muscle unloading. Taking the above-mentioned into account, the present work was dedicated to the study of the effects of HS, sciatic nerve transection or its Caps treatment, as well as their combination upon Cer and Chol amounts, membrane properties (lipid raft integrity and lipid ordering/asymmetry), and Bax/Bcl-2 immunoexpression in rat soleus muscle.

## 2. Results

### 2.1. SMase Immunoexpression in the Soleus Muscle of Control (Sham-Operated Rats) and Denervated Animals

**Figure 1:**
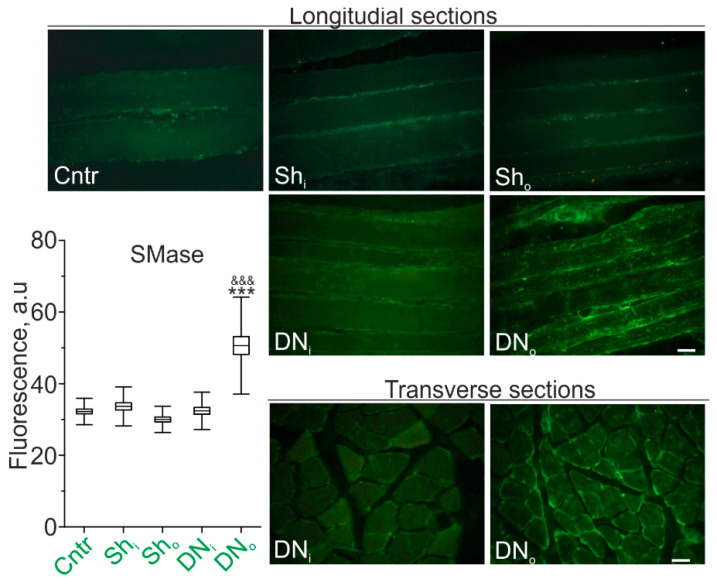
SMase is upregulated in denervated rat soleus muscle. Expression of immunoreactive SMase in sections of soleus muscle fibers of the control (Cntr), unilaterally sham-operated (Sh) and unilaterally denervated (DN) rats. Sh_i_ and Sh_o_—muscles from the intact and sham-operated limb; DN_i_ and DN_o_—muscles from the intact and denervated limb. Shown longitudinal (Cntr, Sh, DN) and transverse (DN) sections. Scale bars—10 μm. Left: quantification of fluorescence (mean; box range—SEM; whiskers—SD); N = 6 animals for each group. *** *p* < 0.001—in comparison to the intact limb; ^&&&^ *p* < 0.001 in comparison to the sham-operated control. *p* was evaluated by a two-way ANOVA followed by Bonferroni post hoc test.

Previously, we found marked upregulation of SMases accompanied by increase in Cer levels in skeletal muscle after 12h HS [5,14]. Here, in control and sham operated control rats, the immunoreactive staining of SMase in longitudinal muscle sections was revealed as a weak fluorescence in sarcolemmal and sarcoplasmic regions of muscle fibers (Figure 1). 

SMase in the plasma membrane was expressed in the form of compact clusters distributed over the fibers surface without a visible regular pattern in both sham-operated (Sh_o_) and intact (Sh_i_) limbs. In animals with transected sciatic nerve, the intensity of SMase immunofluorescence was markedly increased by 68.8 ± 8.8 % in the denervated limb in comparison with the control (sham-operated) one (*p* <0.001). The visible diameter of muscle fibers in the denervated muscle was less than that in the intact one.

### 2.2. Immunodetection of Cer on Transverse Sections of Rat Soleus Muscle in HS Combined with Unilateral Denervation or Caps Treatment of Sciatic Nerve

**Figure 2:**
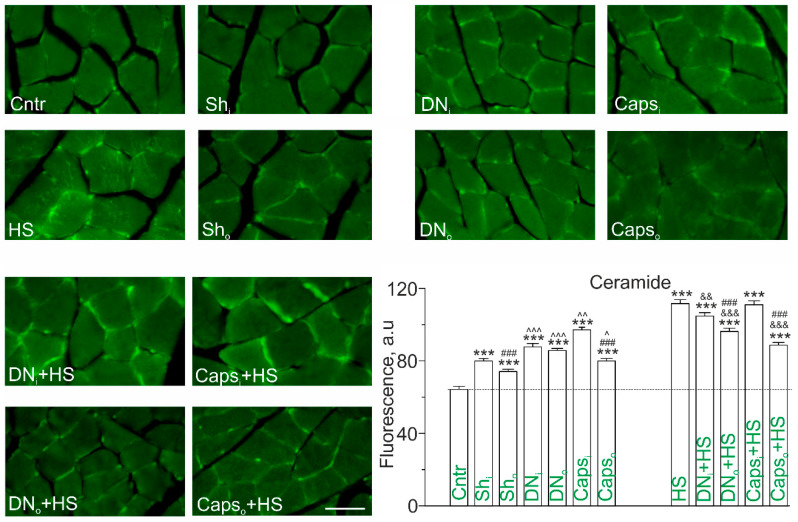
Cer immunoexpression in soleus muscle of HS, unilaterally denervated (DN) or capsaicin (Caps) treated rats. The assay was carried out 5 days after unilateral surgical denervation (DN) or application of Caps on the sciatic nerve. In the experiments with 12-h muscle unloading, DN or Caps deafferentation were performed 5 days before HS. Immunofluorescent images: transverse sections of soleus muscle stained with anti-Cer antibodies. Scale bar—10 μm. Graph: quantification of plasma membrane fluorescence (mean ± SEM); N = 4 in each group. *** *p* < 0.001—differences compared to control; ^ *p* < 0.05, ^^ *p* < 0.01, ^^^ *p* < 0.001—differences compared to sham-operated rats; ^###^ *p* < 0.001—differences between intact and operated limb. ^&&^ *p*< 0.01, ^&&&^ *p*< 0.001. *p* was evaluated by a two-way ANOVA followed by Bonferroni post hoc comparisons. Sh_i_, DN_i_, Caps_i_ and Sh_o_, DN_o_, Caps_o_—intact and operated limb, respectively.

Taking into account a marked upregulation of SMase adjacent to the membrane regions (Figure 1) and observation that 12h HS has the similar effect, immunoexpression of Cer in the plasma membrane was evaluated in conditions of denervation and its combination with HS (Figure 2). 

Unilateral denervation led to considerable increase in Cer immunofluorescence in the membrane regions of muscle fibers from both intact (+44.0 ± 4.9%) and operated (+40.5 ± 3.4%) soleus muscle in comparison to nonoperated control (Figure 2). If comparing to sham-operated rats, denervation increased Cer immunofluorescence by +12.8 ± 2.6% and +18.0 ± 2.5%, in intact and denervated muscle, respectively (Figure 2). The difference between two extremities was not found. Accordingly, the intact muscle at the unilateral denervation also expressed lipid abnormalities. To clarify possible contribution of CSPA input cessation onto muscle Cer accumulation in response to denervation, the chemical inhibition of those afferents was used. Like denervation, Caps application on the sciatic nerve enhanced Cer immunoreactivity in the sarcolemma of both operated and intact muscles. However, Cer staining was higher expressed in intact muscles of unilaterally Caps-treated rats (Figure 2). In comparison with sham operation, Cer immunofluorescence was increased by +26.7 ± 2.2% and +10.27 ± 2.3% in muscle from intact and Caps-treated limb, respectively. The difference between ipsilateral and contralateral limbs was significant.

Hence, denervation can induce Cer accumulation in the sarcolemmal regions probably due to decrease in afferentation via CSPA. Note that if comparing denervation and Caps administration, the latter increased Cer immunofluorescence less markedly in muscle from the operated limb, but more markedly in muscle from the intact limb. 

Consistent with our previous data [14], HS was accompanied by the substantial increase in Cer staining in both the plasma membrane and intracellular compartments of muscle fibers (Figure 2). The magnitude of HS-evoked increment of Cer content was higher than observed in case of denervation or treatment with Caps. Surprisingly, both denervation and Caps treatment visibly attenuated the effects of HS in the soleus muscles from both operated (more markedly) and intact limb. Indeed, the effect was more intensely expressed in the denervated muscle (−13.8% in comparison with HS, *p* < 0.001); in the intact muscle Cer was decreased only by 6 % in comparison with HS (*p* < 0.01). Caps treatment decreased Cer immunoexpression in ipsilateral muscle (−16.2% vs. HS, *p* < 0.001) (Figure 2). 

### 2.3. Cer and Chol Amount in Soleus Muscle Homogenates of Hindlimb Suspended and Unilaterally Denervated Rats

**Figure 3:**
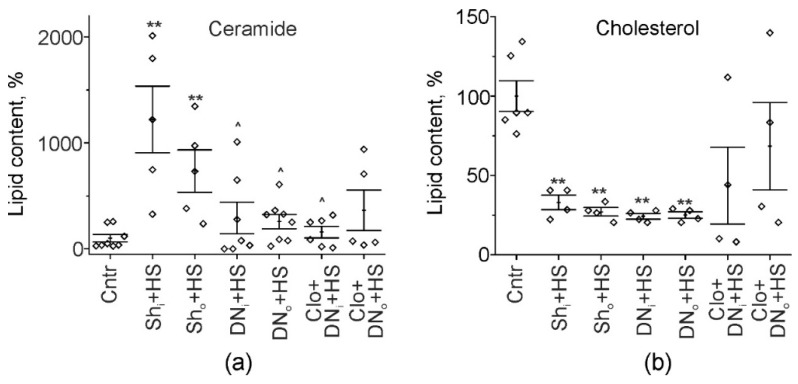
Cer and Chol amount in soleus muscle homogenates. (**a,b**) Quantifications of Cer and Chol content (in %). Shown data for control (Cntr), sham-operated suspended (Sh+HS), unilaterally denervated suspended (DN+HS) and clomipramine-pretreated unilaterally denervated suspended (Clo+DN+HS) rats. Sh_i_ and Sh_o_—muscles from the intact and sham-operated limb; DN_i_ and DN_o_—muscles from the intact and denervated limb. Data are represented as mean ± SEM; diamonds are individual values; N = 5–8 per group. ** *p*< 0.01, —differences compared to control (Cntr); ^ *p* < 0.05—differences compared to HS sham operated animals. *p* was evaluated by Kruskal–Wallis and Mann–Whitney tests.

To validate unexpected fact that denervation can attenuate Cer upregulation in response to HS, total Cer content in muscles was evaluated. Previously we have found that 12-h HS markedly increased muscle Cer content in muscle homogenates [5,11]. Similarly, suspension of sham operated animals led to the bilateral increase of Cer amount in soleus muscle in comparison with intact control rats (*p* = 0.002); statistical difference between ipsilateral and contralateral muscle was not found (Figure 3a). In operated suspended rats, the significant drop of Cer was found (−71.4%, *p* = 0.042, in the intact limb, −64.9%, *p* = 0.045, in the denervated one) in comparison with HS. As a result, Cer content did not differ from the control values (*p* = 0.076 and *p* = 0.22, for ipsilateral and contralateral limbs, respectively). Previously, we found that functional inhibitor of aSMase clomipramine prevents HS-induced Cer accumulation in muscle homogenates [5]. Herein, clomipramine did not additionally affect Cer abundance in both denervated and intact muscles of operated suspended animals (158.5 ± 55.3%, *p* = 0.66, and 362.8 ± 191.5%, *p* = 0.94, respectively, in comparison with HS). This finding raises the possibility that denervation itself can decrease aSMase activity, which is a key factor responsible for Cer buildup after HS [5,14].

Unloading caused the changes in Chol amount, which were opposite to those of Cer. Particularly, Chol levels in muscle decreased after 12h-HS [5]. Similarly, muscle Chol substantially dropped in sham-operated suspended animals after 12h HS (−67.0%, *p* = 0.004, in the intact muscle, and −72.9%, *p* = 0.004, in the muscle from the operated leg). In contrast to Cer levels, denervation did not suppress the effect of HS on Chol levels, which remained reduced in both denervated and intact unloaded muscles (Figure 3b). Note that HS-induced decrease in muscle Chol amount in unilaterally denervated animals pretreated with the inhibitor of aSMase was not statistically significant due to high variability of values (*p*˂0.05 vs. both control and DN_i_/DN_o_+HS) (Figure 3b). Therefore, further experiments are needed to access ability of aSMase inhibitor to modulate Chol levels in denervated suspended muscle.

Thus, denervation was able to partially decrease the effect of HS on total Cer levels, but not on Chol levels. The former effect of denervation is developed despite the ability of denervation itself to upregulate SMase and Cer amounts.

### 2.4. Cer in Detergent Resistant Membrane (DRM) Domains of Rat Soleus Muscle in HS Combined with Unilaterally Denervation or Caps Treatment of Sciatic Nerve

Evident uncoupling in effects on Cer levels of the denervation or Caps application by its own and in combination with HS can be result of compartment-specific changes in Cer content under these conditions. Previously, we have found that 12h HS leads to marked increase in Cer levels in lipid raft fraction—DRM from rat soleus muscle [14]. Accordingly, we decided to elucidate the influence of denervation and Caps application on Cer in DRM; whether HS-mediated changes in lipid raft Cer amount are also affected by preceded surgical or chemical manipulations with the nerve. 

**Figure 4:**
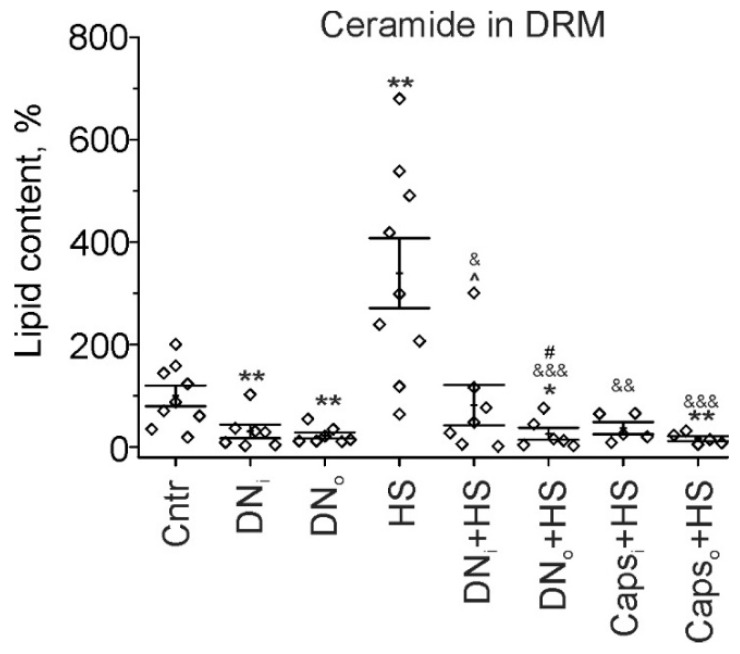
Cer content in DRM fraction. Cer levels in control (Cntr), after unilateral denervation (DN)/Caps treatment (Caps) itself and in combination with HS are shown. Data are represented as mean ± SEM; diamonds are individual values; N = 5−9 per group. * *p*< 0.05, ** *p*< 0.01—differences compared to control; ^&^ *p*< 0.05, ^&&^ *p*< 0.01, ^&&&^ *p*< 0.001—differences compared to HS; ^#^ *p* < 0.05—differences between intact and operated limb, ^—differences between HS and HS+DN_i_. *p* was evaluated by a Kruskal–Wallis and Mann–Whitney tests. DN_i_, Caps_i_ and DN_o_, Caps_o_—intact and operated limb, respectively.

Consistent with our previous study [14], Cer amount in DRM fraction of the unloaded for 12h soleus muscle elevated more than three-fold in comparison with the control rats (+239.3 ± 68.6%, *p* = 0.006). Surprisingly, after unilateral surgical denervation Cer level decreased in DRM of the muscles from both intact and operated limbs (−69.3 ± 13.1%, *p* = 0.005 and −77.3 ± 6.2%, *p* = 0.002 in the intact and denervated muscle, respectively). Denervation completely prevented the effect of HS on Cer content in the raft fraction. Furthermore, Cer level in DRM of denervated muscle decreased below those in the control one (−74.1 ± 11.8%, *p* = 0.012). Similarly, Caps pretreatment of sciatic nerve abolished HS-induced increase in Cer abundance in DRM fraction (Figure 4). Notably, as for denervation, Cer amount was below the control level in the muscle from Caps treated suspended limb (−83.7 ± 4.9%, *p* = 0.004). Thus, despite the ability of denervation to increase total and plasmalemmal Cer, denervation decreased Cer content in lipid raft fraction; as a result, denervation can suppress disuse induced Cer accumulation in the muscle. Inhibition of afferent input with Caps had similar to the denervation effect.

### 2.5. The Effect of Unilateral Denervation Itself and Its Combination with HS on Staining with Lipid Raft Marker

**Figure 5:**
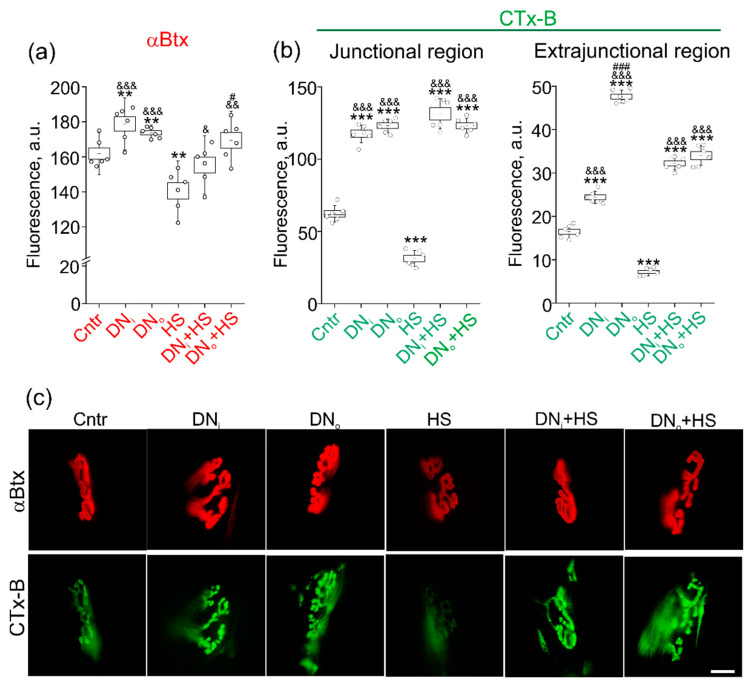
Labeling of AChRs and lipid rafts in muscle. (**a,b**) Quantification of fluorescence (in a.u.) Postsynaptic nAchRs (**a**) or lipid rafts (**b**) were labeled with fluorescent ɑBtx or CTx-B, respectively, in control (Cntr), suspended muscle (HS), after unilateral denervation in intact (DN_i_) and operated (DN_o_) muscle, after unilateral denervation following HS in intact (DN_i_+HS) and operated (DN_o_+HS) muscle. N = 6 animals for each group. (**b**) Left—in junctional (synaptic) region; right—in extrajunctional region. ** *p*< 0.01, *** *p*< 0.001—differences compared control (Cntr); ^&^ *p*< 0.05, ^&&^ *p*< 0.01, ^&&&^ *p*< 0.001—differences compared to HS; ^#^ *p* < 0.05, ^###^ *p*< 0.001—differences between intact and operated limb. *p* was evaluated by a Mann–Whitney U test. (**c**) Representative fluorescent images of junctional regions. Scale bar—10 µm.

Taking into account that denervation selectively decreased Cer content in DRM (considered as analog of membrane rafts in biochemical studies) and prevented HS-induced accumulation of Cer in this membrane fraction, the lipid raft integrity was evaluated by confocal microscopy using a lipid raft marker CTx-B. Previously we found that acute HS disturbs the lipid rafts and decreases staining with fluorescent αBtx (a marker of postsynaptic nAChRs) [4,5,14]. Indeed, HS attenuated labeling with Btx (−13.4 ± 2.9%) and CTxB (−49.7 ± 3.7% in junctional and −56.3 ± 2.2 extrajunctional membranes). In contrast, unilateral denervation slightly increased Btx-labeling (+10.5 ± 2.5% and +7.3 ± 0.8%) in both intact and denervated muscle (Figure 5a). Moreover, unilateral denervation prevented the HS-induced decrease in Btx-staining in intact and denervated muscle (Figure 5a). Similarly, the denervation profoundly increased the labeling of the junctional membrane with CTx-B in intact (+88.6 ± 4.0%) and denervated (+97.6 ± 3.1%) muscle (Figure 5b,c). The denervation completely abolished a decrease in CTx-B labeling due to HS. Furthermore, the combination of the denervation and HS increased a CTx-B signal (110.8 ± 6.9% in intact and +97.7 ± 3.3% in denervated muscle) to the same levels as the denervation alone (Figure 5b,c). Like in junctional region, the denervation itself and its combination with HS led to an increase in CTx-B (denervation and denervation+HS: +48.2 ± 3.6%/+189.2 ± 3.6% and +95.6 ± 3.6%/+106.7 ± 5.7% in intact/denervated muscle) staining in muscle fiber membranes. Lipid rafts are essential for neuromuscular transmission (spontaneous and evoke quantum neurotransmitter release, and nonquantum acetylcholine secretion well as postsynaptic receptor functionality) [6,8,30,41,42,43,44,45,46] and, hence, the increase in lipid raft integrity can be an early compensatory response to denervation-induced disturbance of neuromuscular communications. This compensatory reaction overcomes the effect of acute HS on lipid raft stability. 

Note that the highest enhancement of the CTx-B fluorescence was detected in the extrajunctional regions of denervated muscle. These data indicate that short-term denervation triggers the systemic response that affects both denervated and nondenervated muscles. As a result of this response, the integrity of lipid rafts could be increased in both junctional and extrajunctional regions. The denervation-related response can abolish the lipid raft disrupting action of acute HS as well as prevent a decrease in nAChR packing. Hence, the effects of denervation alone and in combination with HS on lipid rafts reversibly correlate with the changes in Cer levels in raft membrane fraction.

### 2.6. Influence of Unilateral Denervation and Caps-Induced Sensory Denervation on the Effect of HS on Lipid Distribution-Sensitive Dye F2N12S Fluorescence

**Figure 6:**
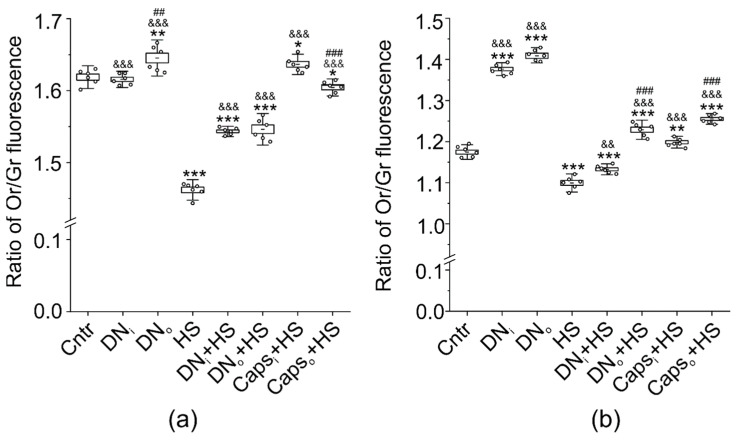
Effects on fluorescence of membrane asymmetry and ordering-sensitive dye F2N12S. (**a**,**b**) The ratio of Or/Gr fluorescence of ratiometric fluorescent probe F2N12S is shown. Junctional (**a**) and extrajunctional membrane (**b**) were labeled with F2N12S in control (Cntr), suspended muscle (HS), after unilateral denervation in intact (DN_i_) and operated (DN_o_) muscle, after unilateral denervation following HS in intact (DN_i_+HS) and operated (DN_o_+HS) muscle, after unilateral application of Caps to the sciatic nerve in intact (Caps_i_+HS) and operated (Caps_o_+HS) muscle. N = 6 animals for each group. * *p*<0.05, ** *p* < 0.01, *** *p* < 0.001—differences compared control (Cntr); ^&&^ *p* < 0.01, ^&&&^ *p* < 0.001—differences compared to HS; ^##^ *p* < 0.01, ^###^ *p* < 0.001—differences between intact and operated limb. *p* was evaluated by a Mann–Whitney U test.

Membrane properties tightly coupled with lipid raft integrity are lipid ordering and asymmetric distribution. There is a possibility that HS can disturb a membrane asymmetry. In agreement with our previous data, a ratio of orange/green (Or/Gr) fluorescence of lipid-distribution sensitive dye F2N12S (4’-N,N-diethylamino-6-(N,N,N-dodecyl-methylamino-sulfopropyl)-methyl -3-hydroxyflavone) was decreased (by −9.7 ± 0.2% in junctional and −6.4 ± 0.5% extrajunctional regions) in response to 12-h HS throughout the membrane (Figure 6a), indicating the reduction of the membrane asymmetry and (or) lipid ordering [47,48]. Unilateral denervation slightly increased the Or/Gr ratio (+1.6 ± 0.4%) in junctional region of the denervated (but not intact) muscle. At the same time, the denervation suppressed the effect of HS on F2N12S fluorescence in both denervated and intact muscles; values of Or/Gr ratio in intact and denervated muscle were between the values in control and after HS. 

Remarkably, the application of Caps [28,49] to the sciatic nerve completely or mostly prevented the HS-mediated reduction of Or/Gr ratio in the junctional region of muscles from the intact or operated limb, respectively (Figure 6a). In muscle fiber membranes (extrajunctional region), changes in F2N12S fluorescence had similar directions (Figure 6b). The denervation significantly increased the Or/Gr ratio in extrajunctional regions of both intact (by+17.2 ± 0.4%) and denervated (by+19.7 ± 0.4%) muscle. In addition, the HS-induced decrease in Or/Gr ratio in extrajunctional region was reduced in intact and prevented in denervated muscle. Finally, Caps application on the sciatic nerve abolished the decrease in Or/Gr ratio in response to HS in intact and operated muscle (Figure 6b). Hence, short-term denervation can increase a membrane asymmetry and (or) ordering in both intact and denervated muscle. This effect is especially pronounced in the extrajunctional region of both intact and denervated muscles, suggesting the systemic response and high sensitivity of the extrajunctional membrane to “trophic” influence deriving from the innervation. Furthermore, the denervation suppressed the HS-induced membrane disturbance in junctional and extrajunctional regions. One of the possible mechanisms by which denervation can elicit the systemic reaction is a cessation of afferent nerve activity. Consistent with this assumption, the unilateral blockage of Caps-sensitive afferents in sciatic nerve prevented the loss of membrane asymmetry (ordering) in response to HS. Furthermore, the effect of Caps on the HS-induced sarcolemmal alterations was expressed more intensely than that of the denervation.

### 2.7. Study of Bax and Bcl-2 in Disused Soleus Muscles of Rats with Unilateral Surgical Denervation or Caps Treatment of the Sciatic Nerve

**Figure 7:**
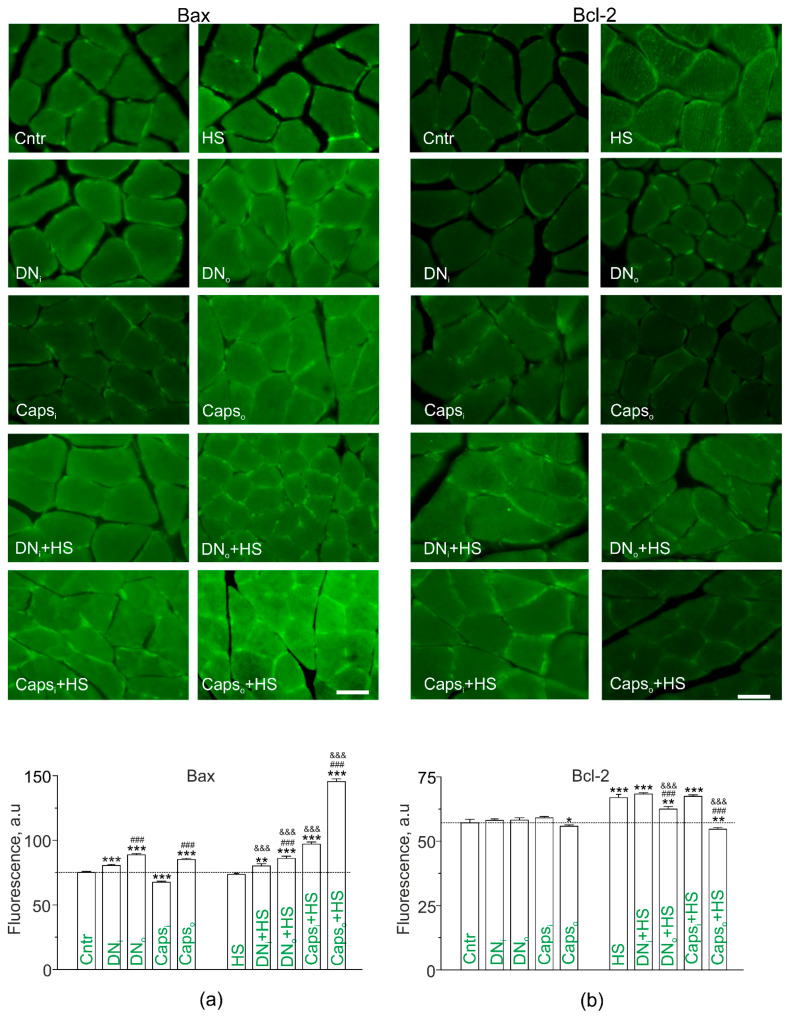
Bax and Bcl-2 immunoexpression. Left (**a**) Bax immune fluorescence is enhanced on longitudinal sections of both denervated (DN_o_) and nondenervated (DN_i_) soleus muscle, but oppositely changed in the muscles from the limbs of rats with unilateral Caps application on the sciatic nerve (Caps_i_—intact and Caps_o_—operated leg). Bax in soleus muscles taken from operated or nonoperated legs of hindlimb suspended rats. Right (**b**) Bcl-2 immune fluorescence is not significantly changed in denervated (DN_o_) and nondenervated (DN_i_) soleus muscle, and slightly decreases in Caps-treated (Caps_o_) muscle. Bcl-2 is upregulated in 12-h disused soleus muscles; Bcl-2 staining is lower in the limbs of suspended with surgically denervated or Caps-treated sciatic nerve animals. Left and right, scale bars—10 µm. (**a,b**) Quantification of fluorescence (mean ± SEM); * *p*< 0.05, ** *p*< 0.01, *** *p*< 0.001—differences compared to control; ^&&&^ *p*< 0.001—differences compared to HS; ^###^ *p*< 0.001—differences between intact and operated limb. *p* was evaluated by a two-way ANOVA followed by Bonferroni post hoc test. N = 4 for each group.

Loss of lipid raft integrity, lipid ordering and asymmetry as well as Cer accumulation can decrease cell viability, leading to destructive alterations in muscle fibers. To assess such changes, immunoexpression of apoptotic-related proteins, proapoptotic Bax (Figure 7a) and protective Bcl-2 (Figure 7b), was evaluated. Bax immunofluorescence was slightly enhanced on transverse sections of both denervated (DN_o_;+18.1%) and nondenervated (DN_i_; +7.0%) soleus muscle. Unilateral Caps application on the sciatic nerve decreased the Bax fluorescence in the intact muscle (−10.1%), but increased it in the operated muscle (+13.3%). The difference between intact and operated limbs was significant for both denervation and Caps treatment (Figure 7a). Interestingly, HS did not modify the fluorescent signal from Bax. Furthermore, HS had no marked effect on denervation-induced slight enhancement of Bax fluorescence, but markedly augmented Caps-mediated increase in Bax levels in the operated limb (in these conditions, Bax signal increased by +93.5% vs. control). Additionally, Caps increased Bax immunoexpression in the intact muscle (+29.1% vs. control).

Changes in immunoexpression of Bcl-2 had another pattern (Figure 7b). Denervation did not affect Bcl-2 fluorescence compared to the control muscle. Caps treatment led to a slight decrease in Bcl-2 immunofluorescence only in the muscles from operated limbs (−2.3%). HS itself increased the immunoexpression of Bcl-2 (+17.1%), and this effect of HS was suppressed by denervation and Caps pretreatment selectively in the muscles from operated limbs. Furthermore, Bcl-2 fluorescence was decreased in muscle from operated limb pretreated with Caps below the control level (−4.4%), whereas its staining in the operated limb remained higher than the control value (+18.0%).

Thus, denervation as well as inhibition of CSPA lead to upregulation of proapoptotic Bax in affected muscles and these can be enhanced by HS in case of Caps action. At the same time, compensatory upregulation of antiapoptotic Bcl-2 in response to HS can be weakened by denervation and Caps in the operated limb. Note that sham operation did not markedly change Bcl-2 immunofluorescent staining in muscle from both intact (60.0 ± 0.5 a.u.; n = 4) and operated (57.7 ± 0.6 a.u.; n = 4) limbs compared to the control (57.1+−1.2 a.u.).

## 3. Discussion

The proven fact is that, in condition of muscle unloading, postural muscles are most predisposed to atrophy [50,51]. Accordingly, the interest of many scientists working in this field is often focused on the changes occurring in postural muscles like soleus. Cer accumulation is an important event in many pathological cascades related with postural muscle dysfunctions [16,52]. Earlier, we have found that 12h HS is accompanied by the increase in SMase and Cer abundance in the unloaded soleus muscle of rat and mice [5,11,14,15]. Here, we showed that the similar effect is due to surgical denervation. These are consistent with upregulation of Cer and glucosylCer synthase after 3–7 days of denervation in mice [20,21]. At the same time, denervation markedly decreased the levels of Cer in raft fraction. This was accompanied by enhancement of junctional and extrajunctional membrane labeling with lipid raft marker, CTxB, suggesting increase in lipid raft integrity. Along the same lines, denervation affected fluorescence of ratiometric probe F2N12S, pointing to raise of lipid ordering and (or) asymmetry. Both these properties positively correlate with lipid raft formation. A possible reason for increase in lipid raft stability can be profound reduction of Cer in raft membrane after denervation. Indeed, since Cer can displace Chol from rafts, it can lead to disruption in normal lipid raft dynamics [18]. Short-term HS can increase Cer levels in raft fraction and decrease lipid raft integrity, whereas inhibition of acid SMase reduced HS-induced accumulation of Cer in rafts as well as disruption of lipid rafts and lipid asymmetry [5,14]. Accordingly, HS and denervation have some similarities, specifically increase in SMase and total Cer content, but HS and denervation regulate Cer levels in raft fraction and lipid raft integrity in the opposite manner. It should be noted that effects of unilateral denervation were expressed in both denervated and intact muscle, excluding increased SMase abundance that was observed only in the denervated muscle. This indicates that systemic response can operate in response to denervation (but not sham operation).

Importantly, preliminary denervation markedly suppressed the effects of HS on Cer levels in homogenates and raft fraction as well as on lipid raft stability and lipid ordering/asymmetry. At the same time, denervation did not modify the ability of HS to decrease Chol levels. This suggests that denervation can modulate the membrane effects of HS mainly via reduction of Cer in raft fraction. The mechanism by which denervation may attenuate Cer deposition in lipid rafts is unknown, but it seems to be independent on acid SMase activity. 

It is established that the muscle atrophy may be associated with loss of support afferentation [53], which also occurs at denervation. To gain insight into the mechanism of the denervation influence, partial chemical deafferentation of the muscle by Caps treatment of the sciatic nerve [28] was applied. Like denervation, the deafferentation led to upregulation of total Cer in soleus muscle and decreased HS-induced Cer accumulation in membrane, including raft fraction. Additionally, Caps application completely prevented disruption of lipid asymmetry/ordering due to HS. Remarkably, the effects of Caps and denervation had a similar degree. Hence, partial lack of afferent activity can be a key contributor to alteration in Cer metabolism and membrane properties in response to denervation. Importantly, muscle unloading due to HS and disturbance of nerve fiber function due to denervation or deafferentation had opposite early effects on Cer in raft fraction and membrane properties. This may be a reflection of triggering different Cer-dependent cascades which shift the steady-state level of Cer in membrane microdomains and, hence, integrity of the microdomains. Another possibility is that a strong compensatory response operates in case of denervation and deafferentation. 

Expression of apoptotic-related proteins can be sensitive to changes in membrane properties and Cer accumulation [54,55]. Immunochemical staining showed that the unilateral denervation as well as unilateral deafferentation slightly increased expression of proapoptotic Bax protein in the muscle from the affected limb; in the intact muscle the effect was smaller or inverted, respectively. Immunolabeling antiapoptotic Bcl-2 protein was not sensitive to denervation, only Caps treatment slightly decreased Bcl-2 levels. These data suggest that at initial step in denervation and deafferentation only negligibly altered apoptotic proteins in apoptosis-promoting direction. HS itself slightly increased Bcl-2 expression without affecting Bax level. These changes in Bcl-2 may reflect the beginning compensatory response. Interestingly, denervation had the same effects on Bax expression in suspended muscle, and decreased HS-induced increase in Bcl-2. Deafferentation of CSPA much stronger increased Bax in combination with HS, and decreased Bcl-2 in disused muscle. These results suggest that apoptotic protein response is unregulated due to combination of HS with denervation and especially CSPA deafferentation. Accordingly, despite decrease in Cer in rafts and increased lipid raft integrity, denervation and deafferentation promotes apoptotic related changes in combination with muscle disuse. This uncoupling raises a possibility that separate Cer- and raft-independent processes control expression of apoptotic proteins under these conditions. 

## 4. Materials and Methods 

### 4.1. Animals 

Experiments were carried out on male Wistar rats (180–230 g). The study conforms to the Guide for the Care and Use of Laboratory Animals (NIH Publication No. 85–23, revised 1996) and the European Convention for the Protection of Vertebrate Animals Used for Experimental and Other Scientific Purposes (Council of Europe No. 123, Strasbourg, 1985). The experimental protocol met the requirements of the EU Directive 2010/63/EU and was approved by the Bioethics Committee of Izhevsk State Medical Academy (Protocol #501/1 from 6.09.2016).

To study the effects of muscle unloading, denervation and chemical deafferentation, we used the following experimental approaches in randomly established groups of animals: (1) absolute control; (2) unilateral transection of the sciatic nerve; (3) unilateral application of 50mM Caps (in isotonic saline) on the sciatic nerve; (4) unilateral sham operation; (5) and (6) 12-h hindlimb unloading with or without sham operation; (7) and (8) 12-h hindlimb unloading with the preliminary denervation or sciatic Caps treatment; (9) hindlimb unloading of unilaterally operated animals with clomipramine pretreatment.

All operations were made 5 days before unloading in animals anesthetized with 0.1 mL of Zoletil 50 (Virbac, France), at a dose of 10mg/kg. Unilateral transection of the sciatic nerve was performed in the mid-thigh region. The surgical site was subjected to the standard procedure of aseptic processing before and after the operation. Sciatic nerve was transected and 5mm of it was removed. Caps treatment of the sciatic nerve was performed according to [56]. Briefly, small sterile pledget of cotton wool soaked in a 50 mM Caps solution was applied to the nerve for 15 min and then removed. All procedures were performed with the described precautionary measures to avoid the Caps effect upon the surrounding tissues. In sham-operated rats only surgical procedures without the nerve treatment were performed. After the operation, animals were placed into individual cages in the standard vivarium environment with 12 h light/dark cycle and ad libitum access to food and water. The inhibitor of acid SMase clomipramine (Anafranil, Novartis Pharma AG) at high dose (1.25 mg/g body weight) was administered intramuscularly (in isotonic saline, volume 0.2 mL) every day, 5 days before the experiment with muscle unloading. This protocol of clomipramine administration has been shown to be effective in our previous studies [5,14]. 

Unloading was reproduced using tail-suspension model according to Il’in-Novikov [57] and Morey-Holton and Globus [1]. 

After the end of the experiments, rats were anesthetized with 0.1 mL of Zoletil 50 and underwent special preparation for the immune fluorescent study (see below). For biochemical study, soleus muscles were harvested, weighed, quickly frozen in liquid nitrogen, and stored at −80 °C. 

### 4.2. Biochemical Study

Cer and Chol levels were assessed in the homogenates of muscle tissue and DRM (for Cer only). To assay Cer amount, high performance thin layer chromatography on HPTLC Silica gel 60 F_254_ plates (Merck, Germany) was used as described previously [21]. Chol in muscle homogenates was estimated as described previously [5].

Briefly, DRM were isolated from the homogenates by ultracentrifugation in sucrose gradient according to Radeva and Sharom [58]. The samples of muscle tissue (10 mg) were homogenized in 1 mL of lysing TBS buffer (1% Triton X-100 in 25 mM Tris/HCl + 140 mM NaCl + protease/phosphatase inhibitors, pH 7.5). The homogenates were incubated at +4 °C for 30 min. The obtained lysates were mixed with 2 mL of 60% sucrose in TBS (25 mM Tris/HCl + 140 mM NaCl, pH 7.5), then sucrose in TBS was added layer-by-layer in order of 1 mL of 30% solution and 1 mL of 5% one. The samples were centrifuged at 300,000g for 3 h at +4° C, and then 0.6–1.0 mL of the top fraction was collected and analyzed.

Lipids were extracted from the homogenates and DRM fractions with Folch reagent (chlorophorm:methanol, 2:1) [59], processed as described previously [60]; after that, the lipid chloroform extracts (0.1 mL) were spotted on Silica gel plates and developed in the butanol:acetic acid:water, 3:1:1 solvent system according to [61]. The equivalent volume of Cer solution in chloroform (Avanti polar lipids, Alabaster, AL, USA) was used as standard. The lipid spots were imaged by iodine vapor. Semiquantitative analysis of Cer was made by video-densitometer (Sorbfil, Russia) at UV light (254 nm) with Sorbfil TLC Videodensitometer software (Sorbpolymer, Russia). The calculation of Cer amount was performed using the means of the standard Cer samples.

Ganglioside GM1 in isolated DRM containing fraction was used as a raft marker. For GM1 detection, chloroform extracts prepared from the DRM containing fraction were developed on HPTLC Silica gel 60 F254 plates (Merck, Germany) with GM1 standard (Avanti polar lipids, Alabaster, AL, USA) in propanol:water 7:3 solvent system as described earlier [59,60].

### 4.3. Immunofluorescence Study 

After the end of the experiments, animals were anesthetized and perfused with phosphate-buffered saline (PBS in mM: 3.2 NaH2PO4, 0.5 K2HPO4, 1.3 KCl, 135 NaCl, pH 7.4) through the ascending aorta. The next step was the perfusion with the solution of 4% parapharmaldehyde in PBS. Then the muscles were postfixed in the same fixative for 2 h, transferred to a 30% sucrose solution for a day and frozen on dry ice. Cer and SMase were studied in the serial longitudinal and transverse muscle sections (14 µm) made by HM525 NX Cryostat (Thermo Fisher Scientific, Waltham, MA, USA). The sections were mounted on the Superfrost Plus slides (Thermo Fisher Scientific, Waltham, MA, USA). Immunofluorescent staining was performed as described previously [14]. Muscle sections were incubated with anti-Cer monoclonal antibodies (mouse IgG, 1:300, Enzo Life Sciences, Farmingdale, NY, USA) or anti-SMase antibodies (mouse IgG, 1:300, Abcam), and then with anti-mouse biotinylated antibodies (goat IgG, 1:200, Abcam), both for 24 h at room temperature. After washing in PBS, mixture of mouse streptavidin-conjugated antibodies (Abcam) with goat anti-mouse (FITC) IgG (1:100, Abcam) was added and the samples were incubated overnight. To detect Bax and Bcl-2 fluorescence, anti-Bcl-2 and anti-Bax antibodies and Alexa Fluor 488 conjugated secondary antibodies (Abcam) were used. The analysis of images was made using the Canon PowerShot 600 photo attachment combined with a Nikon Eclipse E200 microscope (Tokyo, Japan) and performed with Image-Pro Plus 6.0 and Image-Pro Insight software (Media Cybernetics, USA). Cer was studied in sarcolemmal regions of muscle fibers. The intensity of Cer immunofluorescence was determined by digitalized images (image format 2880 * 2048 pixels) using Image-Pro Plus 6.0 and Image-Pro Insight software (Media Cybernetics, USA) as follows: small rectangular regions of interest of ≈2000 (100 * 20 pixels) were created and moved with the cursor to the individual area of the sarcolemma [62]. For measurement, every 5th section was taken, and 10 intensity measurements were made (at least 150 measurements per animal). Mean values were presented in arbitrary units of gray color intensity of binary images. No fluorescent labeling of SMase and Cer were observed without addition of the primary antibody (negative controls).

### 4.4. Confocal Fluorescence Microscopy

Fluorescence was detected using an upright microscope (OlympusBX51WI; Olympus Tokyo, Japan) with a confocal Disk Speed Unit and DIC optics. The images were recorded with a LumPlanPF100xw objective and DP71 (Olympus Tokyo, Japan) CCD camera and then analyzed using ImagePro (version 7.0, Media Cybernetics, Bethesda, MD, USA). Staining with rhodamine-conjugated α-bungarotoxin (α-Btx; 100 ng/mL, T1175T, ThermoFisher, Waltham, MA, USA), a specific dye for postsynaptic nicotinic acetylcholine receptors (nAChRs), was used to define the junctional region. The fluorescence was estimated as the mean intensity in arbitrary units from pixels in the regions of interest. Analysis of junctional fluorescence was performed in the region labeled with α-Btx. Extrajunctional signal was calculated for an area (≈200 μm2) of plasmalemma outside of the α-Btx-staining region [4]. For fluorescent experiments, we recorded the signals from 15–30 neuromuscular junctions and muscle fibers in each animal.

Lipid rafts were labeled with cholera toxin B subunit (CTx-B) conjugated to Alexa Fluor 488 (Molecular Probes). CTx-B specifically binds with GM1 ganglioside patches, mainly residing in the lipid rafts [63]. The muscles were exposed to a physiological solution with CTxB (1 μg/mL for 15 min. Then the muscles were washed for 30 min and fluorescence was detected [46]. α-Btx was added to the bath solution simultaneously with CTx-B. CTx-B/α-Btx fluorescence was excited by light of 488/555 nm and the emission was recorded using a bandpass filter 505–545/610–650 nm.

F2N12S (A35137T, ThermoFisher, Waltham, MA, USA) is a violet excitable ratiometric probe for the detection of the loss of the plasma membrane asymmetry and ordering (Deschenes et al., 2018; Petrov et al., 2020). The preparations were exposed toF2N12S (0.2 µM) for 5 min and then washed with a physiological solution for 10 min. After that, the F2N12S signal was recorded within 1/3 h. The fluorescence was excited by a 405/15 nm and emission was detected at 530/30 nm (green channel) and 585/30 nm (orange channel). The ratio of Or/Gr fluorescence was estimated. A membrane disturbance due to the loss of membrane asymmetry and (or) ordering leads to a decrease in the ratio.

### 4.5. Statistics

Data are presented as mean ± SEM (or both SEM and SD are shown). The sample size (n, number of animals) is shown in each figure and figure legend. Statistical significance was estimated using a Kruskal–Wallis test, Mann–Whitney U test or two-way ANOVA followed by the Bonferroni post hoc test. Values of *p* < 0.05 were considered significant. Origin Pro (Northampton, MA, USA) and Statistica 21.0 (StatSoftInc, Tulsa, OK, USA) were used for analysis.

## 5. Conclusions

Denervation can bidirectionally affect Cer metabolism in the muscle. Particularly, it increased total Cer, but decreased Cer availability in raft fraction. In addition, denervation decreased HS-induced Cer accumulation in nonraft and raft membranes. The effect of denervation on Cer amount in raft inversely correlated with lipid raft integrity; denervation increased raft stability as well as prevented HS-mediated lipid raft disruption in junctional and extrajunctional membranes. Deafferentation with Caps mimics the effects of denervation itself and in combination with HS, pointing to CSPA as an essential element for supporting Cer homeostasis in muscle. The detailed mechanisms of the denervation/deafferentation-driven changes in muscle Cer homeostasis are still to be elucidated. 

## Data Availability

Data are contained within the article and the additional data supporting the findings of this study are available upon request.

## Abbreviations

HS	Hindlimb suspension
CSPA	Capsaicin-sensitive primary afferents
aSMase	Acid sphingomyelinase
Caps	Capsaicin
Cer	Ceramide
Chol	Cholesterol
DN	Denervation
DRM	Detergent resistant membrane fraction
CTx-B	Cholera toxin B subunit
α-Btx	α-bungarotoxin
TRPV1	Transient receptor potential cation channel subfamily V member 1
